# Dialogical Consciousness and Descriptive Experience Sampling: Implications for the Study of Intrapersonal Communication in Sport

**DOI:** 10.3389/fpsyg.2019.00653

**Published:** 2019-04-03

**Authors:** Judy L. Van Raalte, Andrew Vincent, Yani L. Dickens

**Affiliations:** ^1^Department of Psychology, Springfield College, Springfield, MA, United States; ^2^College of Health Sciences, Wuhan Sports University, Wuhan, China; ^3^Counseling Center, SUNY Oneonta, Oneonta, NY, United States; ^4^Counseling Services, University of Nevada, Reno, NV, United States

**Keywords:** athlete, inner experience, open-beginninged methods, presupposition, self-talk

Inner experience and intrapersonal communication research in sport psychology has been largely dominated by a focus on self-talk, which has typically been examined using retrospective self-report measures. Although the existing self-talk literature has addressed aspects of athlete's inner experience, attempts to extend the theoretical scope of intrapersonal communication in sport has been limited by an adherence to linear, causal models of self-talk, as well as by methodological challenges associated with assessing inner experience. The purpose of this paper is to present theoretical and methodological approaches that can be used for further understanding of intrapersonal communication and inner experience in sport. The paper begins with a brief history of sport self-talk theory and research. Next, a discussion of dialogical self (Hermans et al., 1992; Hermans and Hermans-Konopka, 2010) and dialogical consciousness (Larrain and Haye, 2012; Haye and Larrain, 2013) as they relate to sport self-talk theory is presented. Descriptive Experience Sampling (DES), a promising method for exploring inner experience and self-talk in sport is described. We conclude with suggestions related to integrating dialogical theories and DES into the study of intrapersonal communication in sport.

## History of Self-Talk in Sport Psychology

Examining the origins and history of self-talk research in sport psychology provides important insight into strengths and limitations of the literature. Early sport psychology self-talk research primarily involved linear experimental designs that assessed the effects of assigned self-talk on laboratory-based motor learning and motor performance tasks (Landers, 1995). These experimental approaches required self-talk phrases to be categorized, so that hypotheses about how types of self-talk affect learning and performance could be tested. Although linear, causal theories can provide insight related to the effects of self-talk on certain tasks, it is not possible to answer questions such as “How do athletes experience their own self-talk?” “What is the purpose of self-talk in sport?” and “How does self-talk work?” through categorization and experimental testing alone.

The self-talk literature was subsequently shaped by cognitive and cognitive behavioral theories (CBT) of Ellis (1957) and Beck (1975), which focused on self-talk as emblematic of deeply held “core beliefs” related to self-esteem, confidence, self-concept, and self-efficacy. Although cognitive behavioral paradigms advanced the application of mental skills interventions, such theoretical approaches were limited by their conceptualization of the self as autonomous, unitary, and self-contained (Hermans and Hermans-Konopka, 2010). For instance, the assumption that an athlete's critical self-statement reflects low self-esteem leaves little room for the experience of inner conflict (an athlete who oscillates between positive and negative self-concept) or self-talk that echoes the voice of some important other (an athlete hearing a coach saying “that's not good enough” in their head). Researchers who consider self-talk in a broader paradigmatic context and apply methods that circumvent the limitations of retrospective self-report, may inspire new inquiry and advance understanding of inner experience and self-talk.

## Expanding Theory in Intrapersonal Communication: Dialogical Consciousness

Theories from discursive psychology, especially ideas about dialogical self (Hermans et al., 1992; Hermans and Hermans-Konopka, 2010) and dialogical consciousness (Larrain and Haye, 2012; Haye and Larrain, 2013), provide alternative perspectives with potential for expanding current theory, research, and practice in sport psychology. Dialogical theories of self are based on philosophical assumptions of constructivism, which view the self as multifaceted, contextual, and created through interaction with the social world (Hermans et al., 1992; Hermans and Hermans-Konopka, 2010). Perhaps the most notable feature of theories of dialogical consciousness is that key aspects of inner experience are viewed as taking place in the form of a dynamic conversation that is polyphonic, consisting of many “voices” (Hermans et al., 1992; Larrain and Haye, 2012). These voices, which can be based in language, emotion, or other forms of experience, reflect different viewpoints, perspectives, or positions that might occur to a person (Puchalska-Wasyl, 2016). For example these voices might take the form of internalized I-positions that reflect different versions of self (e.g., ideal self, undesired self, real self), internalized interlocutors who represent external figures such as a coach, a close friend, or a therapist, or norms or rules that have been internalized from culture and society (Hermans and Hermans-Konopka, 2010; Puchalska-Wasyl, 2016).

Ideas pertaining to dialogical consciousness were introduced to the sport psychology self-talk literature via the sport-specific model of self-talk, which raises questions pertaining to inner discourse such as “If we already know everything we know, then why do we talk to ourselves?” and “What are we doing when we engage in self-talk?” (Van Raalte et al., 2016, pp. 140–141). Although answers to these questions cannot be understood using linear, causal models that focus on self-talk categorization, they can be addressed through the lens of dialogical self whereby intrapersonal communication is not about messages being sent and received by a singular self but rather a conversation between internalized positions taking place in the society of the mind (Hermans and Hermans-Konopka, 2010). For instance, an athlete who misses a pass may have self-talk such as “not good enough, you have to make that play” and “no worries, you can do it.” If we focus solely on the content, we lose a chance to gain understanding of that athlete's internal world where the first statement may reflect the internalized voice of a critical coach or parent, and the latter may reflect the internalized voice of a mentor or a fan.

Understanding intrapersonal communication in this way opens additional avenues for research, some of which are currently under study in the area of dialogical consciousness but missing from sport psychology. For instance, Hermans (2003) has discussed the importance of power differential between I-positions and interlocutors, suggesting that certain voices are likely to be more influential in consciousness by being more dominant in internal dialogue. In sport psychology, practitioners and researchers would benefit from better understanding which internal voices are dominant and passive and how intentionally used self-talk interacts with athletes' dominant and passive internal voices and performance.

Integrative and confrontational dialogue types present a second avenue for exploration. Integrative internal dialogues move toward synthesis and solution between internalized voices as existing positions come together as part of the construction of a new position, whereas confrontational internal dialogues accentuate difference and result in cognitive dissonance (Hermans and Hermans-Konopka, 2010; Puchalska-Wasyl, 2016, 2017). Intrapersonal communication that takes place between an athlete's inner critic and inner fan could serve as an example of this. In a confrontational dialogue, one position becomes dominant while the other is silenced; this might result in self-talk such as “ignore that positive talk, you are playing like garbage.” Oppositely, an integrative dialogue would move toward a position that includes both “inner critic” and “inner fan” and may result in self-talk such as “you can finish this game strong, but let's work on that in practice next week.” Exploring the extent to which integrative and confrontational dialogues occur for athletes and the ways these different types of dialogues shape athlete experiences could prove useful in understanding intrapersonal communication in sport, especially given the nature of existing applied interventions such as thought stopping and thought replacement, which employ confrontational approaches designed to silence unwanted voices in internal dialogue (Hardy and Oliver, 2014).

The connection between self, culture, and social context is a key feature of dialogical self theory, as internal dialogue is seen as a reflection of both individual experience and larger cultural forces (Hermans, 2003; Hermans and Hermans-Konopka, 2010). Viewing internal dialogue as being inextricably interconnected with the social context has important implications for self-talk in sport and could provide several avenues for future study. For instance, a given internalized position may be an internalization of a prominent cultural narrative or, in the case of sport, some aspect of team culture. This connection between social context, culture, and the internal world of an athlete stands in contrast to traditional causal, linear, category-focused, information-processing views of sport self-talk and provides a theoretical lens through which cultural differences in self-talk can be understood. Integrating theories of dialogical consciousness into existing theories of intrapersonal communication in sport can also direct applied and research attention to racism, sexism, and other oppressive forces that may be manifested as voices that play out in the internal dialogue of athlete consciousness. One of the major challenges associated with these dialogical concepts pertains to their assessment. Standardized self-report questionnaires are limited in capturing athletes' experiences related to dialogical processes.

## Exploring Inner Experience: The Descriptive Experience Sampling (DES) Method

Self-talk research in sport has been constrained by the ways self-talk is studied (Hardy and Jones, 1994; Brinthaupt et al., 2015). Self-report questionnaires have traditionally served as primary sources of self-talk data, despite concerns about their validity (Van Raalte et al., 2014; Van Raalte and Vincent, 2017; Thibodeaux and Winsler, 2018), extensive evidence that these and other retrospective observations are unreliable (e.g., Brewer et al., 1991; Wells and Loftus, 2003), and the fact that recalling inner events is problematic (Hurlburt and Melancon, 1987; Koriat and Bjork, 2005). Approaches that improve upon existing methods have occasionally been used in sport and exercise psychology research, such as think-aloud methods (Fuhrer, 1985; McPherson, 1999; Whitehead et al., 2015), Ecological Momentary Assessment (EMA; Biddle et al., 2009), and the Experience Sampling Method (ESM; Cerin et al., 2001). Although each of these methods sample inner experience *during* sport performance, each has limitations (Dickens et al., 2018). One method that overcomes many of these shortcomings and is well-suited to the exploration of the dialogical self, dialogical consciousness, and the discursive nature of athlete's inner experiences is DES.

DES is a method that uses a random beeper to directly sample “pristine” inner experience contemporaneously and directly, circumventing many of the limitations of self-report measures and retrospection. DES is “open-beginninged,” open-ended, and uses focused non-leading questions like “what was your inner experience, if any, at the moment of the beep” to direct participants to real-time, momentary experience. Whereas, standardized questionnaires, EMA, and ESM are often influenced by the theory of inner experience that they are designed to measure, DES brackets presuppositions to prevent experimenter expectancies from contaminating observed inner experience. DES also offers several methodological improvements that yield high fidelity samples of inner experience. For example, DES includes collections of random representative samples; intensive training to help participants observe and report inner experience; and extensive collaboration with participants around investigating their inner experience through video-recorded interviews within 24 h of sample collection. DES studies have shown high inter-observer reliability (Hurlburt and Heavey, 2002), DES has been validated with Functional Magnetic Resonance Imaging (fMRI) (Kühn et al., 2014), and DES has been shown to be feasible during sport performance (Dickens et al., 2018). The major cost of implementing DES is the quantity-for-quality tradeoff. DES is labor-intensive, requiring 5–10 h of interview time per participant (Hurlburt and Akhter, 2006; McKelvie, 2019).

DES researchers suggest that DES advances understanding of actual momentary inner experience, often yielding unique contributions. For instance, although many have presumed that self-talk is pervasive, if not ubiquitous, in activities such as silent reading or sport performance, DES research has shown that inner experience typically consists of five frequent phenomena (5FP) (Kühn et al., 2014) including inner speaking, inner seeing, sensory awareness, feeling, and unsymbolized thinking. Inner speaking is self-talk spoken silently to oneself, inner seeing is visual imagery, and sensory awareness includes bodily sensation (e.g., pain, tension, hunger), and feeling is emotion (e.g., anxiety, anger, joy). Unsymbolized thinking is a seldom recognized but explicit thought process that takes place without the presence of words or images (see Hurlburt and Akhter, 2008) and occurs about as frequently as the more well-known 5FP (Lapping-Carr and Heavey, 2017). DES research suggests that inner experience is idiosyncratic since inner experiences outside of the 5FP can and do occur, including being in a flow state and completely absorbed in an activity (Lapping-Carr and Heavey, 2017) and having no inner experience occurring at the moment of the beep (Hurlburt and Schwitzgebel, 2011). In a sport context, Dickens et al. (2018) found that inner experience during golf performance included all 5FP, speaking aloud and inner speaking both occurred during golf, self-talk was a frequent but not the predominant inner experience, inner-speaking self-talk was 6 times as frequent as speaking aloud self-talk, and effortful, intentional use of self-talk (i.e., System 2 self-talk) was rare. Also, some participants experienced no self-talk, and one participant reported no inner experience in over half of their samples, illustrating the idiosyncratic nature of inner experience during sport performance.

## Future Directions

Taken together, theories of dialogical self, dialogical consciousness, and DES challenge assumptions and inspire new theorizing and research in the area of intrapersonal communication and inner experience in sport. Considering athlete experience as dialogical allows us to move beyond CBT cause-effect paradigms that focus on categorization of self-talk and explore possible theories related to I-positions and interlocutors, power dynamics, and confrontational vs. integrative inner-dialogue types. DES provides the tools necessary for precise empirical assessment of these theoretical ideas and can provide insights related to self-talk. Indeed, DES research has already shown that self-talk is a less prevalent aspect of inner experience than previously suggested in the sport psychology literature (Dickens et al., 2018). Together, theories of dialogical self, dialogical consciousness, and DES have the potential to advance theoretical and practical knowledge by validating previous findings and/or uncovering new findings.

## Author Contributions

All three authors developed and contributed to this work. AV developed ideas related to dialogical self and dialogical consciousness. YD developed ideas related to Descriptive Experience Sampling (DES).

### Conflict of Interest Statement

The authors declare that the research was conducted in the absence of any commercial or financial relationships that could be construed as a potential conflict of interest.
